# New Signaling Chemicals Spur Worms to Seek Company

**DOI:** 10.1371/journal.pbio.1001240

**Published:** 2012-01-10

**Authors:** Janelle Weaver

**Affiliations:** Freelance Science Writer, Glenwood Springs, Colorado, United States of America

**Figure pbio-1001240-g001:**
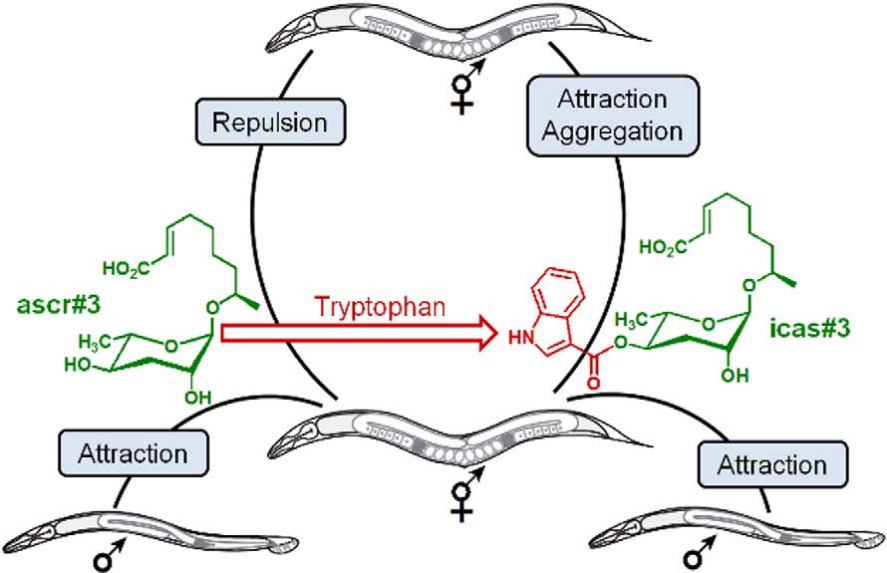
The non-indole ascaroside ascr#3 (a small molecule lacking the compound indole, in green) attracts males but repulses hermaphrodites. Adding the tryptophan-derived indole carboxy moiety (red) to ascr#3 produces icas#3, a potent hermaphrodite attractant.

[Fig pbio-1001240-g001]You may not think much of wriggling roundworms, but for decades, one species of small see-through soil dwellers has engrossed scientists interested in studying a range of behaviors, from sleep to addiction. These well-studied nematodes (*Caenorhabditis elegans*) also display highly variable social habits, with some strains preferring to eat alone while others gorge on bacteria as a group.

The foraging styles of this species are thought to be regulated by small molecules called ascarosides, but these chemical signals aren't one-size-fits-all. They entice males but either repulse or elicit no response in hermaphrodites, which make up the vast majority of the population. Because pheromones that strongly attract hermaphrodites had not been identified, chemical biologist Frank Schroeder of Cornell University and his team suspected the existence of an unknown family of signaling molecules that stimulate nematode aggregation during feeding.

In this month's issue of *PLoS Biology*, these researchers have revealed a new set of potent small molecules—indole ascarosides—that promote aggregation in social and solitary hermaphrodites. The findings suggest that these worms possess a highly evolved chemical language for coordinating social behavior.

To search for novel signaling molecules in *C. elegans*, Schroeder and his team compared metabolites from wild-type organisms with those from mutants lacking the protein DAF-22, which is required for the biosynthesis of all known small-molecule pheromones in this species. They identified several new substances—derived from the amino acid tryptophan in the nematode diet—that are structurally similar to ascarosides, except they contain the compound indole.

Because of the chemical difference between indole and non-indole ascarosides, the team investigated whether they have different effects on behavior. In contrast to repulsive non-indole ascarosides, the indole-containing compounds attracted solitary and social hermaphrodites. Moreover, exposure to a small amount of indole ascarosides caused a strong increase in nematode aggregation.

The researchers then tested which neurons are crucial for these behavioral effects. They found that indole ascarosides still attracted hermaphrodites without RMG neurons, but not worms lacking either ASK neurons or their downstream targets, AIA neurons. These results contrast with previous findings showing that non-indole ascarosides produce their effects by activating ASK and RMG neurons. Thus, these two types of pheromones act on partially non-overlapping neural circuits to alter group behavior in distinct ways.

Finally, the researchers examined how these chemicals interact to regulate worm movements. When they mixed indole ascarosides with a large amount of non-indole ascarosides, they found that the blend attracted hermaphrodites at low concentrations but repelled them at high concentrations, which are common in crowded conditions. Although the newly identified substances may normally lure worms to food sources, this pattern could be reversed by non-indole ascarosides to reduce competition when population densities are high.

The authors suggest that the worms may precisely adjust the chemical blend to control aggregation as a function of the amount of food. By generating a diverse array of chemical signals tailored to environmental conditions, these nematodes display an unexpected level of complexity in their social communication.

**Srinivasan J, von Reuss SH, Bose N, Zaslaver A, Mahanti P, et al. (2012) A Modular Library of Small Molecule Signals Regulates Social Behaviors in** ***Caenorhabditis elegans*** **. doi:10.1371/journal.pbio.1001237**

